# Using Machine Learning Methods in Identifying Genes Associated with COVID-19 in Cardiomyocytes and Cardiac Vascular Endothelial Cells

**DOI:** 10.3390/life13041011

**Published:** 2023-04-14

**Authors:** Yaochen Xu, Qinglan Ma, Jingxin Ren, Lei Chen, Wei Guo, Kaiyan Feng, Zhenbing Zeng, Tao Huang, Yudong Cai

**Affiliations:** 1 Department of Mathematics, School of Sciences, Shanghai University, Shanghai 200444, China; xuyaochen@shu.edu.cn; 2 School of Life Sciences, Shanghai University, Shanghai 200444, China; mql1117@shu.edu.cn (Q.M.); ssdrg@shu.edu.cn (J.R.); 3 College of Information Engineering, Shanghai Maritime University, Shanghai 201306, China; lchen@shmtu.edu.cn; 4 Key Laboratory of Stem Cell Biology, Shanghai Jiao Tong University School of Medicine (SJTUSM) & Shanghai Institutes for Biological Sciences (SIBS), Chinese Academy of Sciences (CAS), Shanghai 200030, China; gw_1992@sjtu.edu.cn; 5 Department of Computer Science, Guangdong AIB Polytechnic College, Guangzhou 510507, China; kyfeng@gdaib.edu.cn; 6 Bio-Med Big Data Center, CAS Key Laboratory of Computational Biology, Shanghai Institute of Nutrition and Health, University of Chinese Academy of Sciences, Chinese Academy of Sciences, Shanghai 200031, China; huangtao@sibs.ac.cn; 7 CAS Key Laboratory of Tissue Microenvironment and Tumor, Shanghai Institute of Nutrition and Health, University of Chinese Academy of Sciences, Chinese Academy of Sciences, Shanghai 200031, China

**Keywords:** COVID-19, cardiovascular disease, cardiomyocytes, vascular endothelial cell, machine learning

## Abstract

Corona Virus Disease 2019 (COVID-19) not only causes respiratory system damage, but also imposes strain on the cardiovascular system. Vascular endothelial cells and cardiomyocytes play an important role in cardiac function. The aberrant expression of genes in vascular endothelial cells and cardiomyocytes can lead to cardiovascular diseases. In this study, we sought to explain the influence of respiratory syndrome coronavirus 2 (SARS-CoV-2) infection on the gene expression levels of vascular endothelial cells and cardiomyocytes. We designed an advanced machine learning-based workflow to analyze the gene expression profile data of vascular endothelial cells and cardiomyocytes from patients with COVID-19 and healthy controls. An incremental feature selection method with a decision tree was used in building efficient classifiers and summarizing quantitative classification genes and rules. Some key genes, such as MALAT1, MT-CO1, and CD36, were extracted, which exert important effects on cardiac function, from the gene expression matrix of 104,182 cardiomyocytes, including 12,007 cells from patients with COVID-19 and 92,175 cells from healthy controls, and 22,438 vascular endothelial cells, including 10,812 cells from patients with COVID-19 and 11,626 cells from healthy controls. The findings reported in this study may provide insights into the effect of COVID-19 on cardiac cells and further explain the pathogenesis of COVID-19, and they may facilitate the identification of potential therapeutic targets.

## 1. Introduction

Corona Virus Disease 2019 (COVID-19), caused by respiratory syndrome coronavirus 2 (SARS-CoV-2) [1,2,3], was first declared a pandemic on 11 March 2020 due to its quick global spread [4,5]. As of 23 July 2022, over 569 million cases have been reported worldwide, with over 6.3 million deaths [6]. Fever, sore throat, dry cough, and pneumonia symptoms are the common clinical manifestations of COVID-19 [7], and the majority of patients with COVID-19 initially exhibit upper respiratory symptoms resembling those of the common flu [8]. However, acute respiratory distress syndrome (ARDS), multiorgan failure, and possibly death have been reported in a small percentage of people with SARS-CoV-2 infection [9]. ARDS is the most common complication in patients with COVID-19, especially elderly patients with underlying diseases, such as diabetes [10].

However, COVID-19 not only harms the respiratory system, but also the cardiovascular system [11]. Approximately 20–30% of patients with COVID-19 have presented with cardiovascular problems linked to poor outcomes [12,13]. Specifically, COVID-19 may increase the risk of cardiovascular diseases, such as myocarditis, arrhythmia, atherosclerosis, and coronary artery disease [14,15,16,17]. For example, viral myocarditis is a type of heart disease possibly caused by COVID-19. Three potential pathogenic mechanisms are widely recognized: (a) SARS-CoV-2 directly damages cardiomyocytes through ACE2 receptors expressed in the heart [18], (b) COVID-19-induced cytokine storm leads to cardiomyocyte hypoxia and apoptosis [19,20], and (c) COVID-19 causes interferon-mediated systemic inflammation that damages cardiomyocytes [13,21]. Some drugs used to treat COVID-19 can cause heart damage, especially antivirals [15]. Therefore, experts in the field are particularly interested in cardiovascular damage caused by SARS-CoV-2 infection and the potential mechanisms associated with cardiac damage.

Vascular endothelial cells and cardiomyocytes are closely related to heart function and cardiovascular diseases. Vascular endothelial cells form the cell lining of arteries, veins, and capillaries [22]; actively control vasoconstriction; and are crucial for controlling immunological response, inflammation, and angiogenesis [23]. Cardiomyocytes are responsible for generating contractile force in the heart and can control the rhythmic beating of the heart [24]. Dysfunction of vascular endothelial cells can heighten the likelihood of developing cardiovascular diseases, such as atherosclerosis [25] and heart failure [26], and can also indirectly result in myocardial dysfunction due to impaired blood flow [27]. Cardiomyocyte dysfunction is associated with several heart diseases, such as cardiac hypertrophy [28], heart failure [29,30], and myocarditis [31].

In this study, we investigated the effect of SARS-CoV-2 infection on gene expression in vascular endothelial cells and cardiomyocytes. The gene expression profiles were retrieved from the GEM database for the cardiomyocytes and vascular endothelial cells of patients with COVID-19 and healthy controls. To build quantitative guidelines for precise prediction, many machine learning algorithms were employed to constitute a computational workflow. The purpose was to identify key genes differentially expressed in the heart after SARS-CoV-2 infection. The features and rules discovered can facilitate the prediction and diagnosis of COVID-19 cardiovascular-related sequelae and aid in understanding the potential pathogenic mechanisms of COVID-19-induced heart damage.

## 2. Materials and Methods

**Figure 1:**
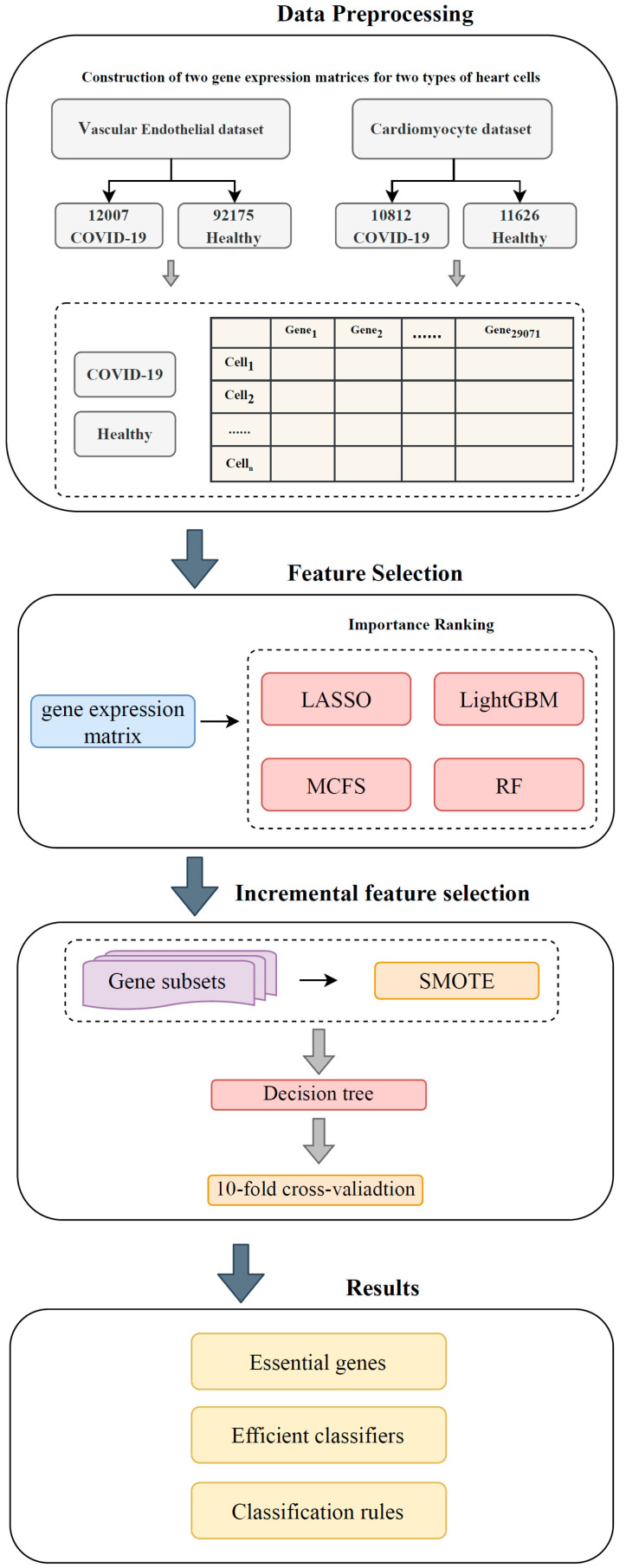
Flow chart of the entire analysis process. Gene profile data for 104,182 cardiomyocytes and 22,438 vascular endothelial cells were analyzed. Each cell contained 29,071 gene expression levels. Genes were ordered according to the degree of correlation with COVID-19 using four feature selection methods. The obtained four ordered gene lists were fed into the incremental feature selection (IFS) method, where decision tree (DT) was employed as the classification algorithm. Finally, the best feature subsets and classification rules were extracted according to the IFS results using DT.

The flow of the machine learning-based workflow designed in this study is shown in Figure 1. Two types of heart cell samples were grouped according to COVID-19 status. The genes that were used to represent cells were ranked according to importance using four methods. Finally, by combining the incremental feature selection (IFS) [32] method and decision tree (DT) [33], some key genes and quantitative classification rules were obtained. This section describes the methods used in each segment.

### 2.1. Data

The current study integrated data from the GEM database [34], which contains the gene expression profiles of cardiac cells from patient with COVID-19 versus healthy controls. The database was downloaded from https://singlecell.broadinstitute.org/single_cell/study/SCP1216 (accessed on 23 May 2022). Cardiomyocytes and vascular endothelial cells in the heart were analyzed because they are closely related to heart function. Cells were grouped according to the cell type, and we obtained two sets of data. The first set contained 104,182 cardiomyocyte samples, including 12,007 COVID-19 cardiomyocytes and 92,175 healthy cardiomyocytes. The second set contained 22,438 vascular endothelial cells, of which 10,812 were COVID-19 vascular endothelial cells and 11,626 were healthy vascular endothelial cells. Each cell was represented by 29,071 gene expression features. In each cell type, COVID-19 and healthy control were deemed labels, which were combined with features to constitute a classification system. Essential features can be obtained by investigating such classification system.

### 2.2. Feature Selection Methods

Each sample contained a large number of gene expression features, but only a small fraction was associated with COVID-19. We analyzed these genes using the following four feature selection methods: least absolute shrinkage and selection operator (LASSO) [35], light gradient boosting machine (LightGBM) [36], Monte Carlo feature selection (MCFS) [37], and random forest (RF) [38], and we ranked them according to their association with COVID-19.

#### 2.2.1. Least Absolute Shrinkage and Selection Operator

In the LASSO algorithm, a first-order penalty function was constructed using the L1 paradigm, where independent variables are the genetic features we input. By applying penalties to the variables with low correlation and small predictive contribution, the corresponding coefficients are reduced to zero, thereby eliminating these unimportant features. Such operation can reduce the data dimension and prevent overfitting. By observing the optimized function, the input features can be sorted according to the absolute values of the coefficients of the features.

#### 2.2.2. Light Gradient Boosting Machine

LightGBM improves the gradient boosting DT algorithm. In a large dataset, it can merge some mutually exclusive features and eliminate those with small gradients, thus achieving data dimensionality reduction and improving efficiency. It uses a leaf-wise strategy when constructing a tree, so it extends branches with high efficiency each time during feature evaluation. The more a feature is involved in building the tree, the more it contributes to the prediction. Thus, features can be ranked according to their occurring times in the constructed DTs.

#### 2.2.3. Monte Carlo Feature Selection

The MCFS algorithm constructs a number of independent DTs. The algorithm randomly selects some features used for constructing the nodes of trees many times. For each feature set, the algorithm randomly selects training data many times. Eventually, p feature subsets are obtained, and for each feature subset, training data are randomly constructed t times, and DTs are constructed. Thus, p×t trees are obtained. The relative importance score (RI) can symbolize the importance of a feature *g*.

(1) $$RI_{g}=\sum_{\tau =1}^{p\times t}{\left(\omega A_{CC}\right)}^{u}\sum_{ng\left(\tau \right)}IG(ng\left(\tau \right)){\left(\frac{no.in\;ng\left(\tau \right)}{no.in\;\tau }\right)}^{v},$$

In the formula, $\omega A_{CC}$ is the weighted accuracy of the tree τ under consideration, $ng\left(\tau \right)$ is a node of the DT whose information gain is denoted as $IG(ng\left(\tau \right))$, $no.in\;ng\left(\tau \right)$ denotes the sample size of $ng\left(\tau \right)$, and no.in τ is the number of samples in the root of τ; u and v are two positive reals weighting the $\omega A_{CC}$ and the ratio $no.in\;ng\left(\tau \right)/no.in\;\tau$, respectively. In terms of decreasing order of RI scores, features can be ranked in a list.

#### 2.2.4. Random Forest

Permutation feature importance was first introduced for RFs by Breiman [39] in 2001 and was later applied to other models by Fisher et al. [38]. The rationale is easy to explain. If a feature is more important, it leads to a greater increase in prediction error when it is randomized. The importance of features can be measured by the increase in prediction error after feature permutation. If permuting its value does not increase the prediction error, the feature is not important. Based on the increment in prediction error, features can be ranked in a list.

### 2.3. Incremental Feature Selection

Using the above methods, four ranked feature lists were obtained. However, it is still challenging to select which features to participate in classification. Given a classification algorithm, its performance under the selected features should provide good performance. This study used the IFS method [32] to determine such features. It constructs a series of feature subsets from a given feature list. The first subset contains the first 10 features, and then each new subset includes an increment of ten following features in the list. Each of these subsets is used to train the downstream classifier based on a given classification algorithm. The performance of all classifiers is subsequently evaluated using 10-fold cross-validation. Based on the performance of the classifiers, the classifier with best performance can be obtained and the best feature subset used in this classifier is picked up for further investigation.

### 2.4. Synthetic Minority Oversampling Technique

Looking at the data set, we found a disparity in the number of samples in each category. For example, the number of healthy cardiomyocytes was 7.7 times that of COVID-19 cardiomyocytes. The direct use of these data would result in preferences for majority classes. This study used the synthetic minority oversampling technique (SMOTE) [40] to address this issue. For a minority class, the method randomly selects a sample, say *α*, and looks for its *k* closest neighbors of the same class according to the Euclidean distance. Sample *b* is randomly selected among these *k* neighbors, and a point is randomly identified on the line segment connecting *α* and *b*. This point is taken as a new sample of the minority class. This process is repeated to generate enough new samples for the minority class so that the number of samples of each class in the dataset is balanced.

### 2.5. Decision Tree

The operation of the IFS method requires a classification algorithm. Here, we selected the DT algorithm [33], which constructs a tree structure with a trunk and leaves. The trunk represents the process of classifying the samples and has several nodes, which contain tests on features. Samples are assigned to different trunks according to the tests. Eventually, the samples reach the leaves, which represent the categories. The DT algorithm is a classical white-box algorithm; that is, its decision process is transparent and can suggest meaningful clues to facilitate the elucidation of the effect of COVID-19 on the expression levels of genes. These clues are included in a group of quantitative classification rules, which can be extracted from the constructed trees.

### 2.6. Performance Evaluation

In IFS method, many classifiers were constructed and evaluated using 10-fold cross-validation [41]. The F1 measure was taken as the key metric in measuring the performance of the classifiers [42,43,44,45,46,47,48]. The calculation procedure was as follows:

(2) $$Precision=\frac{TP}{TP+FP},$$

(3) $$Recall=\frac{TP}{TP+FN},$$

(4) $$F1\;measure=\frac{2\times (Recall\times Precision)}{Recall+Precision},$$

where TP is true positive, FP is false positive, and FN is false negative. Classifier performance increases with F1 measure.

We further used accuracy (ACC) and Matthew correlation coefficient (MCC) [49] for reference. MCC indicates the agreement between predicted and observed labels and is balanced when classes have different sample sizes. The performance of a classifier increases with MCC and ACC.

## 3. Results

The gene expression profiles of cardiomyocytes and cardiac vascular endothelial cells from patients with COVID-19 and healthy population were analyzed in this study, as shown in Figure 1. The results obtained in each step are presented in this section.

### 3.1. Results of Feature Ranking and Incremental Feature Selection

**Figure 2:**
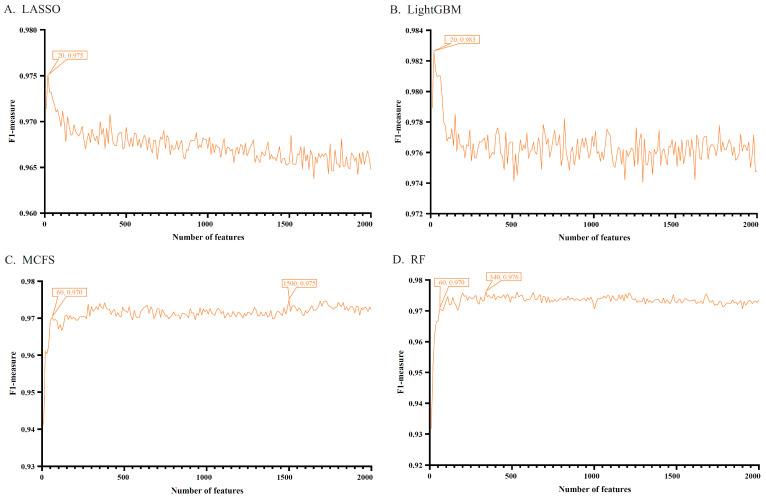
IFS curves for evaluating the performance of the decision tree under different numbers of top features in four lists. Four feature lists were generated by four feature selection methods, corresponding to four IFS curves (cardiomyocytes). (**A**) IFS curves based on the feature list yielded by LASSO. (**B**) IFS curves based on the feature list yielded by LightGBM. (**C**) IFS curves based on the feature list yielded by MCFS. (**D**) IFS curves based on the feature list yielded by RF.

**Figure 3:**
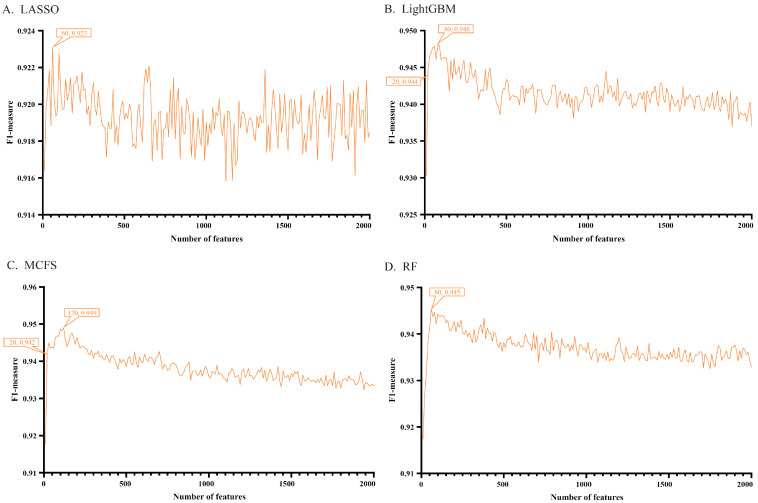
IFS curves for evaluating the performance of the decision tree under different numbers of top features in four lists. Four feature lists were generated by four feature selection methods, corresponding to four IFS curves (vascular endothelial cells). (**A**) IFS curves based on the feature list yielded by LASSO. (**B**) IFS curves based on the feature list yielded by LightGBM. (**C**) IFS curves based on the feature list yielded by MCFS. (**D**) IFS curves based on the feature list yielded by RF.

Each heart cell contained 29,071 gene expression signatures, which were analyzed using LASSO, LightGBM, MCFS, and RF methods. Four ranked feature (gene) lists were obtained, which are provided in Table S1. As only a small number of genes would be significantly associated with COVID-19, top 2000 genes in each list were picked up for subsequent analysis. Using the IFS method and the DT algorithm, 200 feature subsets were constructed from each list, thereby inducing 200 DT classifiers. All classifiers were assessed by 10-fold cross-validation. Table S2 provides the evaluation results of all classifiers. To clearly display the performance of classifiers under different feature subsets, IFS curves were plotted using F1 measure as the vertical coordinate and the number of genes (features) as the horizontal coordinate. The results are shown in Figure 2 and Figure 3.

On the cardiomyocyte dataset, four IFS curves on four feature lists are illustrated in Figure 2. It can be observed that the DT classifier using the first 20 genes in the list yielded by LightGBM showed the best performance, with an F1 measure of 0.983. In addition, it provided high ACC and MCC (0.996 and 0.980, respectively, see Table 1). The best DT classifiers on other three feature lists also showed excellent performance. The highest F1 measures were 0.975 (LASSO), 0.975 (MCFS), and 0.976 (RF) when first 20, 1500, and 340 genes were used in the corresponding lists, respectively. Their detailed performance is listed in Table 1.

On the dataset of vascular endothelial cells, four IFS curves are shown in Figure 3. The DT classifier using the first 120 genes in the list yielded by MCFS showed the highest performance (F1 measure of 0.949) and the best ACC and MCC values (0.951 and 0.902, respectively) were obtained on the same set of genes, which are listed in Table 1. Similarly, the best DT classifiers on the lists yielded by LASSO, LightGBM, and RF also provided high performance, which generated the F1 measure values of 0.923, 0.948, and 0.945, respectively, by using the first 60, 80, and 60 genes in the corresponding lists.

According to the above results, the best DT classifiers on each feature list all gave high performance, meaning that they can be useful tools to identify COVID-19 patients.

**Figure 4:**
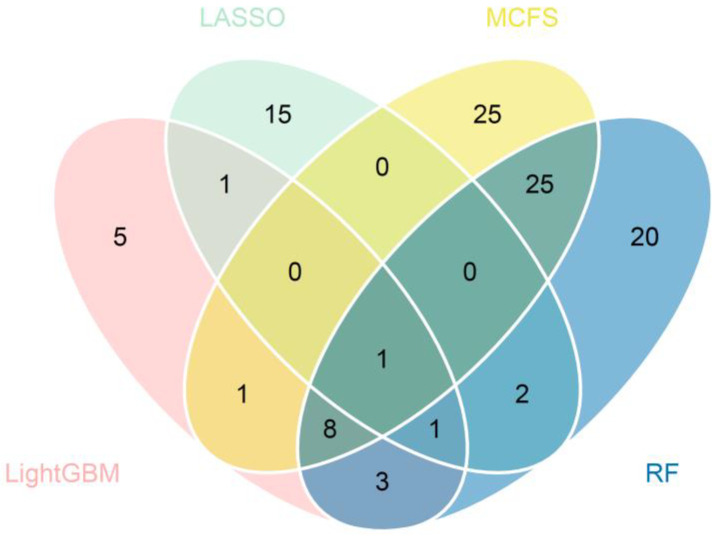
Venn diagram of four subsets of important genes identified by LASSO, LightGBM, MCFS, and RF (cardiomyocytes). Overlapping circles indicate the number of genes identified by multiple feature selection methods.

**Figure 5:**
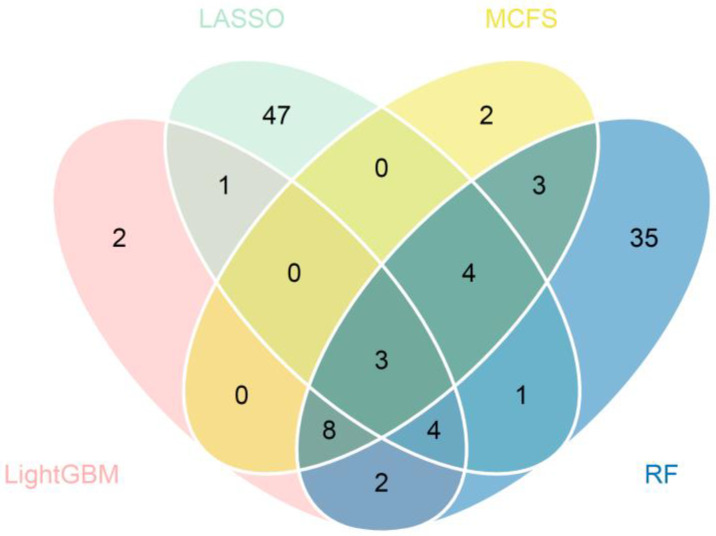
Venn diagram of four subsets of important genes identified by LASSO, LightGBM, MCFS, and RF (vascular endothelial cells). Overlapping circles indicate the number of genes identified by multiple feature selection methods.

For each cell type, important genes were extracted from each feature list, with which DT classifier can yield the best performance. The number of genes is listed in Table 1. However, lots of such genes were extracted from some feature lists. For example, for cardiomyocytes, 1500 genes were accessed from the list yielded by MCFS. In this case, it was not easy to conduct the further investigation. In view of this, the most important genes should be extracted from these genes. After checking the IFS results on two cell types, we selected the top 60 genes from the list yielded by MCFS and top 60 genes from the list yielded by RF for cardiomyocytes, and we selected the top 20 genes from the list yielded by LightGBM and top 20 genes from the list yielded by MCFS for vascular endothelial cells. The F1-measure values of DT classifiers under above features are marked in Figure 2 and Figure 3. Compared with the F1-measure values of the best DT classifiers on the same feature list, they were a little lower. However, the numbers of used features were sharply reduced. Thus, these genes were relatively more important than rest genes. For the important genes selected from other feature lists, it was not necessary to make further selection as only a few genes were picked up. Accordingly, the top 20, 20, 60, and 60 genes in the lists yielded by LASSO, LightGBM, MCFS, and RF for cardiomyocytes, respectively, were selected to find the intersection and a Wayne diagram was plotted, as shown in Figure 4. Similarly, the most important genes (the first 60, 20, 20, and 60 genes of the lists) for vascular endothelial cells were analyzed. The results of the Wayne diagram are shown in Figure 5. As expected, we searched the key genes commonly indicated by these methods and shown to be closely associated with COVID-19. Detailed intersection results are shown in Table S3.

### 3.2. Classification Rules

The DT algorithm is a classical white-box algorithm. It can explicitly show the process of classification and provide interpretable classification clues. These rules may facilitate the analysis of the significance of genes, which may be differentially expressed in patients with COVID-19. By using the best DT classifiers on four feature lists, we summarized four groups of quantitative classification rules for cardiomyocytes and cardiovascular endothelial cells, respectively. The detailed classification rules are provided in Table S4. On one hand, these rules can be used to distinguish patients with COVID-19 from healthy controls. On the other hand, they can clearly display the different expression patterns in cardiomyocytes or cardiovascular endothelial cells between healthy controls and patients with COVID-19. Some rules will be discussed in detail below.

## 4. Discussion

We identified a set of potential signature genes that reveal differential expression associated with COVID-19 in cardiomyocytes and vascular endothelial cells. These genes can be useful in understanding how SARS-CoV-2 infection affects gene expression in cardiac cells. Confirming these genes can enhance the understanding of the pathogenesis of COVID-19-induced cardiovascular diseases, aiding in the clinical diagnosis and treatment of related clinical diseases. Several features and quantitative rules we identified are related to COVID-19-caused cardiovascular diseases according to newly published papers.

### 4.1. Analysis of Gene Features in Cardiac Cells for COVID-19

Based on the machine learning-based workflow, a set of significant genes were identified, which may be differentially expressed in vascular endothelial cells and cardiomyocytes. The genes facilitated the differentiation of patients with COVID-19 from healthy populations. Under pathological conditions, several top genes are involved in distinct biological activities in cardiomyocytes and vascular endothelial cells. Here, we analyzed the first five genes listed in Table 2 for cardiomyocytes and vascular endothelial cells.

The possible impact of the differential expression of identified top features in vascular endothelial cells and cardiomyocytes on the hearts of patients with COVID-19 is discussed below. This discussion may explain the vulnerability of patients with COVID-19 to cardiovascular diseases and may offer direction for clinical diagnosis and treatment.

#### 4.1.1. Qualitative Features in Vascular Endothelial Cells

The first identified feature gene is *MALAT1* (ENSG00000251562), which has a significant impact on the heart given that it positively regulates the proliferation of cardiomyocytes [50] and plays an important role in the regulation of cardiovascular inflammation [51]. Low levels of *MALAT1* lncRNA were discovered in patients with severe COVID-19 [52,53]. Another study suggested that *MALAT1* depletion is responsible for the sepsis inflammatory response by inhibiting the expression of IL-6 and TNF-α and the NF-κB signaling pathway by upregulating miR-150-5p [54]. COVID-19 is associated with proinflammatory cytokine release [55], suggesting that SARS-CoV-2 infection alters the expression of *MALAT1*. Therefore, *MALAT1* can be considered a potential feature for identifying patients with COVID-19. In vascular endothelial cells, the differential expression of *MALAT1* is associated with several cardiovascular diseases, and *MALAT1* is associated with the inflammation and apoptosis of vascular endothelial cells [56], risk of coronary heart disease [57,58], and deep vein thrombosis [59].

The next identified features were *ID1* (ENSG00000125968) and *ID3* (ENSG00000117318). They belong to the inhibitor of DNA binding (ID) family. IDs are required for the formation of the heart [60,61] and skeletal myogenesis [62] and play an important role in angiogenesis [63]. Thus, a potential relationship between IDs and the heart was revealed. A recent study discovered *ID1* differential expression in COVID-19 retest-positive patients [64], demonstrating the potential influence of COVID-19 on ID expression. The beneficial function of IDs in reducing viral replication has been shown in some articles [65,66]. The expression of IDs in vascular endothelial cells may have a potential role in increasing the risk of some cardiovascular diseases. *ID3* expression is involved in the protective process of coronary artery disease [67] and atherosclerosis [60]. Moreover, *ID1* plays an important role in repair after a vascular injury [68].

The next identified gene was *MT-CO1* (ENSG00000198804), which is a cytochrome c oxidase gene and a mitochondrial marker that plays an important role in mitochondrial aerobic respiration [69]. Given that the heart’s driving function depends on ATP produced by aerobic respiration, MT-CO1 is essential for the heart. Several studies have demonstrated the effect of COVID-19 on *MT-CO1* expression. In 2022, researchers found low mRNA levels of *MT-CO1* in patients with COVID-19 [70], and another article noted that MT-CO1 was downregulated in patients with COVID-19 and recovered individuals [71]. The differential expression of *MT-CO1* in vascular endothelial cells due to COVID-19 may contribute to a range of cardiovascular diseases. The inhibition of *MT-CO1* expression may lead to inefficient electron transfer, resulting in the production of reactive oxygen species [72]. The resulting damage to the vascular endothelium has detrimental effects on cardiac function. In addition, MT-CO1 downregulation produces mitochondrial oxidative stress, which may increase the risk of atherosclerosis and coronary artery disease [73]. Thus, the higher risk of cardiovascular disease in patients with COVID-19 is partially explained by the differential expression of the *MT-CO1* gene in vascular endothelial cells [74].

The last identified feature was *EGFL7* (ENSG00000172889). *EGFL7* gene is associated with angiogenesis and is highly expressed in the developing neonatal vasculature [75]. It is upregulated in adults with a vascular injury [76,77]. In 2020, Leng et al. [78] discovered that EGFL7 is downregulated in patients with COVID-19, suggesting that *EGFL7* can be identified as a valid feature for distinguishing patients with COVID-19. The negative consequences that may be caused by *EGFL7* gene differential expression in vascular endothelial cells in the heart have been explored. One study discovered that increasing *EGFL7* expression enhances neoangiogenesis within plaques and promotes the development of atherosclerosis [79]. Moreover, *EGFL7* knockdown induces cardiac dysfunction and fibrosis [80], and *EGFL7* genetic deletion causes severe vascular defects [81].

#### 4.1.2. Qualitative Features in Cardiomyocytes

The first identified feature gene was *MALAT1* (ENSG00000251562). We have discussed the basic function of the *MALAT1* gene and its differential expression in patients with COVID-19. In contrast to the previous discussion, the COVID-19-induced differential expression of *MALAT1* in cardiomyocytes produced different negative effects on patients’ hearts. Increased *MALAT1* expression enhances cardiomyocyte apoptosis [50,82,83], and *MALAT1* is highly expressed in patients with acute myocardial infarction [84,85]. However, the low expression of *MALAT1* may promote myocardial hypertrophy [86].

The next identified gene was *CD36* (ENSG00000135218). It plays an important role in lipid metabolism, especially in the uptake of long-chain fatty acids, which are the primary source of myocardial energy supply, which explains why *CD36* is so closely related to heart function [87]. *CD36* is differentially expressed in patients with COVID-19 and recovered individuals [88]. Moreover, human primary monocytes infected with SARS-CoV-2 alter the expression of genes related to lipid uptake, such as *CD36* [89]. All these results suggest that COVID-19 may alter *CD36* gene expression. Alterations in *CD36* gene expression may cause some adverse effects on the heart and leads to the disruption of fatty acids or lipid metabolism, resulting in a range of chronic diseases, such as cardiac hypertrophy, heart failure, and cardiac ischemia/reperfusion [90]. The myocardial uptake of long-chain fatty acids is significantly reduced, and glucose utilization is increased in *CD36*-deficient patients [91]. A shift in energy supply accelerates the progression of myocardial hypertrophy to heart failure [92].

The next identified feature was *LARGE1* (ENSG00000133424), which is highly abundant in the heart [93] and involved in the regulation of cellular homeostasis in myocytes [94]. Although no direct evidence has shown that *LARGE1* gene expression is altered in patients with COVID-19, two studies on the Lassa fever virus, which also contains spike proteins, found *LARGE1* gene overexpression [95,96]. Thus, *LARGE1* expression is likely to change as a result of SARS-CoV-2 infection. *LARGE1* overexpression exacerbates muscular dystrophy [97,98]. Given that this disease affects cardiac function and even leads to heart failure [99], the altered expression of *LARGE1* in cardiac myocytes caused by COVID-19 may have an adverse effect on the heart.

The next identified gene was *RYR2* (ENSG00000198626), which is highly expressed in the heart, is involved in action potential regulation in atrial myocytes [100], and positively regulates atrial contractility [101]. These functions indicate the important role of *RYR2* in the heart. Indirect evidence has demonstrated the association between COVID-19 and *RYR2* expression. COVID-19 may cause hypoxia in patients [102], and *RYR2* expression can be significantly reduced upon hypoxic exposure [103]. *RYR2* channels exhibit increased activity in a patient’s brain [104], and alterations in *RYR2* expression may cause a range of negative effects on the heart. Moreover, pathological Ca^2+^ channel remodeling and heart failure progression are linked to *RYR2* [105,106]. Additionally, catecholamine-sensitive ventricular tachycardia is caused by *RYR2* dysfunction [107], and low *RYR2* expression likely causes a comparable range of negative effects.

The last identified feature was *PLCG2*(ENSG00000197943), which is a SARS-CoV-2 infection-related gene associated with immune response [108]. SARS-CoV-2 infection may lead to viral myocarditis, which may be associated with the expression of *PLCG2*. The expression of *PLCG2* transcripts is upregulated in club cells after SARS-CoV-2 infection [109], and *PLCG2* is highly expressed in the kidneys of patients with COVID-19 [108]. Cardiac injury due to COVID-19-induced differential expression of *PLCG2* in cardiomyocytes may be associated with myocarditis. Given that *PLCG2* is involved in multiple immune responses [110,111], its high expression may promote inflammation leading to myocarditis.

### 4.2. Analysis of Decision Rules in Cardiac Cells for COVID-19

As described above, we identified a validated set of genes that may help in qualitatively distinguishing cardiac gene expression samples from patients with COVID-19 from that in uninfected populations by using cardiac gene expression. The ability of some genes in a sample categorization at the transcriptome level can be confirmed by recent studies. In addition, quantitative rules were also established based on the computational workflow, and some representative rules for cardiomyocytes and vascular endothelial cells were selected for in-depth discussion, as listed in Table 3. The rules allowed us to accurately identify patients with COVID-19 according to changes in gene expression in cardiac cells.

We then predicted the potential impact of SARS-CoV-2 infection on patients’ hearts and the likelihood of developing cardiovascular diseases according to parameters differentially expressed in each rule. The pathogenicity analysis of the parameters in the rule is more specific than that in the previous section and is strictly based on the “upregulation” or “downregulation” indicated in the rule.

#### 4.2.1. Quantitative Rules in Vascular Endothelial Cells

The first rule (Rule 0) involves four parameters that help in differentiating a population infected with SARS-CoV-2 and reflecting on the impact of COVID-19 on heart function. The first parameter *MALAT1* (ENSG00000251562) was downregulated in this rule for the identification of patients with COVID-19. In 2020, a study found that *MALAT1* was downregulated in the bronchoalveolar lavage fluids of patients with mild and severe SARS-CoV-2 infections [112]. Another study in 2021 found similar results [113]. *MALAT1* can be considered a reliable parameter for distinguishing patients with COVID-19 despite the fact that *MALAT1* expression levels varied with the severity of COVID-19 and the type of cells tested [52,114]. *MALAT1* is associated with vascular endothelial cell homeostasis [115], and reduced *MALAT1* expression may impede vascular repair after SARS-CoV-2 infection. *MT-CO1* (ENSG00000198804), as a second parameter, was downregulated in vascular endothelial cells after SARS-CoV-2 infection in this rule. In 2022, researchers found reduced mRNA levels of *MT-CO1* in patients with COVID-19 [70], validating this parameter. Another study from 2021 discovered that after SARS-CoV-2 infection, the expression of mitochondrial genes, including the *MT-CO1* gene, was downregulated [116]. Reduced *MT-CO1* expression in this rule may increase the incidence of coronary artery disease and contributes to poor outcomes in patients with coronary artery disease [117,118]. The next parameter in the vascular endothelium was *HIF3A* (ENSG00000124440), which was shown to be downregulated in this rule. In 2021, HIF3A was found to be downregulated within the frontal cortices of patients with COVID-19 [119]. HIF3A is induced by hypoxia [120] and downregulated in the inflammatory states [121], partially explaining the validity of the parameters. However, studies on the potential damage caused by low *HIF3A* expression to the heart are limited. A 2021 publication found that silencing *HIF3A* reduces apoptosis and has a cardioprotective effect [122]. The last parameter in Rule 0 was *SNHG7*(ENSG00000233016), which was predicted to be downregulated in the vascular endothelium of patients with COVID-19. In 2021, researchers found that *SNHG7* lncRNA was upregulated in SARS-CoV-2-infected cells and tissues of patients with COVID-19 [123]. This result is contrary to the prediction of Rule 0. The testing of other tissues, such as the lung tissues, which were more exposed to different quantities of SARS-CoV-2 than the heart, may be the cause of the discrepancy between the results. A recent publication suggested that SNHG7 provides protection against atherosclerosis development [124], suggesting that patients with COVID-19 are more likely to develop atherosclerosis or more severe atherosclerosis. SNHG7 was downregulated in unstable plaques of patients with coronary artery disease [125], suggesting the potential role of its low expression in the regulation of coronary artery disease.

The second rule (Rule 1), which helped us identify healthy people without SARS-CoV-2 infection, contained four parameters. The first parameter was *MALAT1* (ENSG00000251562), whose expression in vascular endothelial cells was consistently high in healthy populations. The downregulation of *MALAT1* in patients with COVID-19 and the potential effects on cardiac health have been discussed. The second parameter, *ID1* (ENSG00000125968), showed consistently high levels in vascular endothelial cells in this rule and was used in distinguishing individuals who were not infected by SARS-CoV-2. The knockdown of *ID1* was found to be beneficial for the replication and transcription of multiple viruses [65,66], partially demonstrating that *ID1* helps in distinguishing uninfected populations. Endocytic activation and angiogenesis are hampered by *ID1* gene inhibition [126], and high levels of *ID1* expression regulate the severity of inflamed tissue injury [127]. Therefore, lowering its expression following SARS-CoV-2 infection may reduce protection against heart damage. The next parameter was *PDLIM5* (ENSG00000163110), which had low levels in the vascular endothelial cells of the SARS-CoV-2-uninfected population. Given the crucial function of *PDLIM5* in repair when the heart is damaged [128,129], low levels of *PDLIM5* in vascular endothelial cells in healthy populations seem understandable. Given that *PDLIM5* is a pro-atherosclerotic gene [130], SARS-CoV-2 infection that results in increased *PDLIM5* expression levels may accelerate atherosclerosis. The next parameter, *PHACTR1* (ENSG00000112137), had low levels in vascular endothelial cells in this rule. It was used in distinguishing uninfected individuals from patients with COVID-19. According to a 2018 study, *PHACTR1* interacts with MRTF-A to mediate inflammation, which may help to explain why uninfected people continue to have low levels of *PHACTR1*. The COVID-19-mediated overexpression of *PHACTR1* may cause a range of cardiovascular diseases, including coronary artery disease [131,132,133] and acute myocardial infarction [133].

#### 4.2.2. Quantitative Rules in Cardiomyocytes

The first rule (Rule 0), with two parameters, facilitated the identification of patients with COVID-19 according to the differential expression of these parameters in cardiomyocytes. The first parameter was *MALAT1* (ENSG00000251562), which showed low expression levels in the cardiomyocytes of patients with COVID-19. Low *MALAT1* expression in cardiomyocytes can cause damage to the hearts of COVID-19 patients. Chen et al. [86] found that *MALAT1* knockout exacerbates angiotensin II-induced cardiac hypertrophy, suggesting that patients with COVID-19 are susceptible to myocardial hypertrophy. As for the second parameter, *TTN* (ENSG00000155657), which was shown downregulated in this rule, was used in distinguishing patients with COVID-19. Kanduc et al. [134] found that titin, a protein expressed by the *TTN* gene, shares 29 pentapeptides with the echinocandin of SARS-CoV-2. This similarity may cause immune cross-reactivity and thus affect *TTN* gene expression. Another publication stated that *TTN* is downregulated after IL-6 treatment [55], implying a corresponding downregulation in patients with COVID-19. *TTN* expression and myocardial function are tightly associated [55,135], and cardiomyocytes with *TTN* gene knockdown show weaker and disordered muscle nodes [136], which may decrease the heart’s ability to contract.

As for the second rule (Rule 1), it contained three parameters for the identification of populations without SARS-CoV-2 infection. The first parameter was *EMC10* (ENSG00000161671), which had low levels in the cardiomyocytes of uninfected individuals. With regard to this gene’s potential impact on the heart, SARS-CoV-2 infection may result in the overexpression of *EMC10*, a gene primarily involved in cardiac repair [134]. As a result, we considered it a potential adaptive repair response. *NEB* (ENSG00000183091), which was demonstrated in this rule to retain low expression levels in the cardiomyocytes of uninfected people, was the parameter used in excluding SARS-CoV-2-infected people. SARS-CoV-2 infection affects *NEB* gene expression and induces an inflammatory response [137], contributing to the exclusion of healthy individuals. Patients with COVID-19 have higher levels of *NEB* expression in their cardiomyocytes. Actin filament length is regulated by *NEB* [138], suggesting that *NEB* overexpression may cause myonodal dysfunction, which in turn results in impaired heart contraction. The last parameter in this rule was *PTGDS* (ENSG00000107317), a gene whose specific expression level in cardiac myocytes helps in distinguishing healthy individuals without SARS-CoV-2 infection. In this rule, the expression of *PTGDS* should be maintained at a high level. In 2021, Haslbauer et al. [139] found that *PTGDS* gene expression was downregulated in patients with COVID-19, suggesting the dysregulation of arachidonic acid metabolism. The heart may be affected by COVID-19-induced decreased expression of *PTGDS*, which is involved in the production of prostaglandin D2 necessary for the survival of cardiomyocytes [140]. Therefore, increased cardiomyocyte apoptosis due to decreased *PTGDS* expression can harm the heart. Furthermore, Zhao et al. [141] revealed that reduced *PTGDS* expression may be a biomarker of myocardial infarction, indicating that COVID-19 patients may have a higher risk of experiencing this condition.

Overall, as we have already indicated, previous studies have validated the acquired rules and supported the top quantitative rules.

### 4.3. Limitations of This Study

In the current study, some essential information (essential genes and classification rules) was extracted from a large gene expression profile on two types of heart cells for COVID-19 patients and healthy controls. Some genes and rules can be confirmed to be related to COVID-19-caused cardiovascular diseases by looking up recent publications. However, solid evidence (through wet experiments) was not provided in this study. Furthermore, lots of genes and rules were output by the machine learning-based workflow. Only a few of them were discussed. Some essential findings may be hidden in the undiscussed part. It is hopeful that the following studies can identify more essential genes and rules.

## 5. Conclusions

To investigate the effect of COVID-19 on cardiac cells, a machine learning-based workflow was designed in this study, which analyzed the gene expression data of two heat cell types from patients with COVID-19 and healthy controls, including cardiomyocytes and cardiac vascular endothelial cells. Some essential genes related to COVID-19 in these cells were accessed, which can be latent biomarkers for identifying COVID-19 patients. On the other hand, effective classification rules were established, indicating the special expression patterns in these cells for COVID-19 patients. Furthermore, some efficient DT classifiers were built in this study, which can be useful tools to diagnose COVID-19 patients. These findings provided a reference for uncovering the mechanism by which COVID-19 damages the heart and suggests some possible therapeutic targets. Unlike traditional drug target studies, which investigate the association between a drug and genes, we believe the drug should target certain cells and the genes within these cell types. The single cell method will revolutionize the drug discovery.

## Figures and Tables

**Table 1:** Performance of the best classifiers constructed for cardiomyocytes and vascular endothelial cells using four lists.

Cell Type	Feature Selection Method	Number of Features	F1 Measure	Matthew Correlation Coefficient	Accuracy
Cardiomyocytes	Least absolute shrinkage and selection operator	20	0.975	0.972	0.994
	Light gradient boosting machine	20	0.983	0.980	0.996
	Monte Carlo feature selection	1500	0.975	0.972	0.994
	Random forest	340	0.976	0.973	0.994
Vascular endothelial cells	Least absolute shrinkage and selection operator	60	0.923	0.851	0.925
	Light gradient boosting machine	80	0.948	0.900	0.950
	Monte Carlo feature selection	120	0.949	0.902	0.951
	Random forest	60	0.945	0.895	0.947

**Table 2:** Top five genes identified by the computational workflow in vascular endothelial cells and cardiomyocytes.

Cell Type	Ensembl ID	Gene Symbol	Description
Vascular endothelial cells	ENSG00000251562	MALAT1	metastasis associated lung adenocarcinoma transcript 1
	ENSG00000125968	ID1	inhibitor of DNA binding 1
	ENSG00000117318	ID3	inhibitor of DNA binding 3
	ENSG00000198804	MT-CO1	mitochondrially encoded cytochrome c oxidase I
	ENSG00000172889	EGFL7	EGF like domain multiple 7
Cardiomyocytes	ENSG00000251562	MALAT1	metastasis associated lung adenocarcinoma transcript 1
	ENSG00000135218	CD36	CD36 molecule
	ENSG00000133424	LARGE1	LARGE xylosyl- and glucuronyltransferase 1
	ENSG00000198626	RYR2	ryanodine receptor 2
	ENSG00000197943	PLCG2	phospholipase C gamma 2

**Table 3:** Representative rules generated in cardiac cells to identify patients with COVID-19.

Cell Type	Rules	Parameters	Predicted Class
Vascular endothelial cells	Rule 0	ENSG00000251562 (MALAT1) ≤ 5.9107	COVID-19 patients
		ENSG00000198804 (MT-CO1) ≤ 1.6645	
		ENSG00000124440 (HIF3A) ≤ 2.2516	
		ENSG00000233016 (SNHG7) ≤ 1.9428	
	Rule 1	ENSG00000251562 (MALAT1) > 6.0103	People without SARS-CoV-2 infection
		ENSG00000125968 (ID1) > 2.7774	
		ENSG00000163110 (PDLIM5) ≤ 0.4622	
		ENSG00000112137 (PHACTR1) ≤ 1.4398	
Cardiomyocytes	Rule 0	ENSG00000251562 (MALAT1) ≤ 5.0148	COVID-19 patients
		ENSG00000155657 (TTN) ≤ 5.0027	
	Rule 1	ENSG00000161671 (EMC10) ≤ 3.9595	People without SARS-CoV-2 infection
		ENSG00000183091 (NEB) ≤ 3.9691	
		ENSG00000107317 (PTGDS) > 0.6654	

## Data Availability

The data presented in this study are openly available in GEM database at https://singlecell.broadinstitute.org/single_cell/study/SCP1216 (accessed on 23 May 2022), reference number [34].

## Supplementary Materials

The following supporting information can be downloaded at: https://www.mdpi.com/article/10.3390/life13041011/s1, Table S1: Feature lists obtained by using LASSO, LightGBM, MCFS, and RF; Table S2: IFS results of decision tree on different feature lists for two cell types; Table S3: Intersection of important genes identified by LASSO, LightGBM, MCFS, and RF for the two cell types. The features identified by 4, 3, 2, and 1 method(s) are shown; Table S4: Classification rules generated by the optimal DT classifiers on different feature lists for two cell types.

## References

[1] Long B., Carius B.M., Chavez S., Liang S.Y., Brady W.J., Koyfman A., Gottlieb M. (2022). Clinical update on COVID-19 for the emergency clinician: Presentation and evaluation. Am. J. Emerg. Med..

[2] Coronavirus Disease (COVID-19)—World Health Organization. https://www.who.int/emergencies/diseases/novel-coronavirus-2019.

[3] Adil M.T., Rahman R., Whitelaw D., Jain V., Al-Taan O., Rashid F., Munasinghe A., Jambulingam P. (2021). SARS-CoV-2 and the pandemic of COVID-19. Postgrad. Med. J..

[4] Chavez S., Long B., Koyfman A., Liang S.Y. (2021). Coronavirus disease (COVID-19): A primer for emergency physicians. Am. J. Emerg. Med..

[5] Raman B., Bluemke D.A., Luscher T.F., Neubauer S. (2022). Long covid: Post-acute sequelae of COVID-19 with a cardiovascular focus. Eur. Heart J..

[6] COVID-19 Map. https://coronavirus.jhu.edu/map.html.

[7] Parasher A. (2021). COVID-19: Current understanding of its pathophysiology, clinical presentation and treatment. Postgrad. Med. J..

[8] Thakur V., Ratho R.K. (2022). Omicron (b.1.1.529): A new SARS-CoV-2 variant of concern mounting worldwide fear. J. Med. Virol..

[9] Song C., Li Z., Li C., Huang M., Liu J., Fang Q., Cao Z., Zhang L., Gao P., Nie W. (2022). SARS-CoV-2: The monster causes COVID-19. Front. Cell. Infect. Microbiol..

[10] Li X., Ma X. (2020). Acute respiratory failure in COVID-19: Is it “typical” ards?. Crit. Care.

[11] Wang D., Hu B., Hu C., Zhu F., Liu X., Zhang J., Wang B., Xiang H., Cheng Z., Xiong Y. (2020). Clinical characteristics of 138 hospitalized patients with 2019 novel coronavirus-infected pneumonia in Wuhan, China. JAMA.

[12] Cizgici A.Y., Zencirkiran Agus H., Yildiz M. (2020). COVID-19 myopericarditis: It should be kept in mind in today’s conditions. Am. J. Emerg. Med..

[13] Sawalha K., Abozenah M., Kadado A.J., Battisha A., Al-Akchar M., Salerno C., Hernandez-Montfort J., Islam A.M. (2021). Systematic review of COVID-19 related myocarditis: Insights on management and outcome. Cardiovasc. Revasc. Med..

[14] Liu J., Deswal A., Khalid U. (2021). COVID-19 myocarditis and long-term heart failure sequelae. Curr. Opin. Cardiol..

[15] Tajbakhsh A., Gheibi Hayat S.M., Taghizadeh H., Akbari A., Inabadi M., Savardashtaki A., Johnston T.P., Sahebkar A. (2021). COVID-19 and cardiac injury: Clinical manifestations, biomarkers, mechanisms, diagnosis, treatment, and follow up. Expert Rev. Anti Infect. Ther..

[16] Chen N., Zhou M., Dong X., Qu J., Gong F., Han Y., Qiu Y., Wang J., Liu Y., Wei Y. (2020). Epidemiological and clinical characteristics of 99 cases of 2019 novel coronavirus pneumonia in wuhan, china: A descriptive study. Lancet.

[17] Siddiq M.M., Chan A.T., Miorin L., Yadaw A.S., Beaumont K.G., Kehrer T., Cupic A., White K.M., Tolentino R.E., Hu B. (2022). Functional effects of cardiomyocyte injury in COVID-19. J. Virol..

[18] Chen L., Li X., Chen M., Feng Y., Xiong C. (2020). The ace2 expression in human heart indicates new potential mechanism of heart injury among patients infected with SARS-CoV-2. Cardiovasc. Res..

[19] Zheng Y.Y., Ma Y.T., Zhang J.Y., Xie X. (2020). COVID-19 and the cardiovascular system. Nat. Rev. Cardiol..

[20] Wei Z.Y., Qian H.Y. (2020). Myocardial injury in patients with COVID-19 pneumonia. Zhonghua Xin Xue Guan Bing Za Zhi.

[21] Channappanavar R., Fehr A.R., Zheng J., Wohlford-Lenane C., Abrahante J.E., Mack M., Sompallae R., McCray P.B., Meyerholz D.K., Perlman S. (2019). Ifn-i response timing relative to virus replication determines mers coronavirus infection outcomes. J. Clin. Investig..

[22] Monahan-Earley R., Dvorak A.M., Aird W.C. (2013). Evolutionary origins of the blood vascular system and endothelium. J. Thromb. Haemost..

[23] Kruger-Genge A., Blocki A., Franke R.P., Jung F. (2019). Vascular endothelial cell biology: An update. Int. J. Mol. Sci..

[24] Sutanto H., Lyon A., Lumens J., Schotten U., Dobrev D., Heijman J. (2020). Cardiomyocyte calcium handling in health and disease: Insights from in vitro and in silico studies. Prog. Biophys. Mol. Biol..

[25] Xu S., Ilyas I., Little P.J., Li H., Kamato D., Zheng X., Luo S., Li Z., Liu P., Han J. (2021). Endothelial dysfunction in atherosclerotic cardiovascular diseases and beyond: From mechanism to pharmacotherapies. Pharmacol. Rev..

[26] Sun B., Meng M., Wei J., Wang S. (2020). Long noncoding rna pvt1 contributes to vascular endothelial cell proliferation via inhibition of mir-190a-5p in diagnostic biomarker evaluation of chronic heart failure. Exp. Ther. Med..

[27] Park J.H., Shin H.H., Rhyu H.S., Kim S.H., Jeon E.S., Lim B.K. (2021). Vascular endothelial integrity affects the severity of enterovirus-mediated cardiomyopathy. Int. J. Mol. Sci..

[28] Nakamura M., Sadoshima J. (2018). Mechanisms of physiological and pathological cardiac hypertrophy. Nat. Rev. Cardiol..

[29] Gao G., Chen W., Yan M., Liu J., Luo H., Wang C., Yang P. (2020). Rapamycin regulates the balance between cardiomyocyte apoptosis and autophagy in chronic heart failure by inhibiting mtor signaling. Int. J. Mol. Med..

[30] Oeing C.U., Pepin M.E., Saul K.B., Agircan A.S., Assenov Y., Merkel T.S., Sedaghat-Hamedani F., Weis T., Meder B., Guan K. (2023). Indirect epigenetic testing identifies a diagnostic signature of cardiomyocyte DNA methylation in heart failure. Basic Res. Cardiol..

[31] Caforio A.L., Baritussio A., Basso C., Marcolongo R. (2022). Clinically suspected and biopsy-proven myocarditis temporally associated with SARS-CoV-2 infection. Annu. Rev. Med..

[32] Liu H., Setiono R. (1998). Incremental feature selection. Appl. Intell..

[33] Safavian S.R., Landgrebe D. (1991). A survey of decision tree classifier methodology. IEEE Trans. Syst. Man Cybern..

[34] Delorey T.M., Ziegler C.G.K., Heimberg G., Normand R., Yang Y., Segerstolpe Å., Abbondanza D., Fleming S.J., Subramanian A., Montoro D.T. (2021). COVID-19 tissue atlases reveal SARS-CoV-2 pathology and cellular targets. Nature.

[35] Ranstam J., Cook J. (2018). Lasso regression. J. Br. Surg..

[36] Ke G., Meng Q., Finley T., Wang T., Chen W., Ma W., Ye Q., Liu T.-Y. (2017). Lightgbm: A highly efficient gradient boosting decision tree. Adv. Neural Inf. Process. Syst..

[37] Dramiński M., Koronacki J. (2018). Rmcfs: An r package for monte carlo feature selection and interdependency discovery. J. Stat. Softw..

[38] Fisher A., Rudin C., Dominici F. (2019). All models are wrong, but many are useful: Learning a variable’s importance by studying an entire class of prediction models simultaneously. J. Mach. Learn. Res..

[39] Breiman L. (2001). Random forests. Mach. Learn..

[40] Chawla N.V., Bowyer K.W., Hall L.O., Kegelmeyer W.P. (2002). Smote: Synthetic minority over-sampling technique. J. Artif. Intell. Res..

[41] Kohavi R. (1995). A Study of Cross-Validation and Bootstrap for Accuracy Estimation and Model Selection. Int. Jt. Conf. Artif. Intell..

[42] Powers D. (2011). Evaluation: From precision, recall and f-measure to roc., informedness, markedness & correlation. J. Mach. Learn. Technol..

[43] Huang F., Fu M., Li J., Chen L., Feng K., Huang T., Cai Y.-D. (2023). Analysis and prediction of protein stability based on interaction network, gene ontology, and kegg pathway enrichment scores. BBA-Proteins Proteom.

[44] Huang F., Ma Q., Ren J., Li J., Wang F., Huang T., Cai Y.-D. (2023). Identification of smoking associated transcriptome aberration in blood with machine learning methods. BioMed Res. Int..

[45] Wu C., Chen L. (2023). A model with deep analysis on a large drug network for drug classification. Math. Biosci. Eng..

[46] Wang H., Chen L. (2023). Pmptce-hnea: Predicting metabolic pathway types of chemicals and enzymes with a heterogeneous network embedding algorithm. Curr. Bioinform..

[47] Tang S., Chen L. (2022). Iatc-nfmlp: Identifying classes of anatomical therapeutic chemicals based on drug networks, fingerprints and multilayer perceptron. Curr. Bioinform..

[48] Ren J., Zhang Y., Guo W., Feng K., Yuan Y., Huang T., Cai Y.-D. (2023). Identification of genes associated with the impairment of olfactory and gustatory functions in COVID-19 via machine-learning methods. Life.

[49] Matthews B.W. (1975). Comparison of the predicted and observed secondary structure of t4 phage lysozyme. Biochim. Biophys. Acta.

[50] Li L., Wang Q., Yuan Z., Chen A., Liu Z., Wang Z., Li H. (2018). Lncrna-malat1 promotes cpc proliferation and migration in hypoxia by up-regulation of jmjd6 via sponging mir-125. Biochem. Biophys. Res. Commun..

[51] Martens C.R., Bansal S.S., Accornero F. (2019). Cardiovascular inflammation: Rna takes the lead. J. Mol. Cell. Cardiol..

[52] Abbasi-Kolli M., Sadri Nahand J., Kiani S.J., Khanaliha K., Khatami A., Taghizadieh M., Torkamani A.R., Babakhaniyan K., Bokharaei-Salim F. (2022). The expression patterns of malat-1, neat-1, thril, and mir-155-5p in the acute to the post-acute phase of COVID-19 disease. Braz. J. Infect. Dis..

[53] Devadoss D., Acharya A., Manevski M., Pandey K., Thurman M., Nair M., Borchert G.M., Mirsaeidi M., Byrareddy N., Chand H.S. (2021). Severe COVID-19 patients and a 3d airway tissue model of SARS-CoV-2 infection express high levels of airway mucins and associated immunomodulatory long noncoding rnas. TP105 Basic Mechanisms of Lung Infections: From SARS-CoV-2 to Influenza.

[54] Wei S., Liu Q. (2019). Long Noncoding rna Malat1 Modulates Sepsis-Induced Cardiac Inflammation through the mir-150-5p/nf-κb axis. Int. J. Clin. Exp. Pathol..

[55] Cantu N., Vyavahare S., Kumar S., Chen J., Kolhe R., Isales C.M., Hamrick M., Fulzele S. (2022). Synergistic effects of multiple factors involved in COVID-19-dependent muscle loss. Aging Dis..

[56] Huang K., Yu X., Yu Y., Zhang L., Cen Y., Chu J. (2020). Long noncoding rna malat1 promotes high glucose-induced inflammation and apoptosis of vascular endothelial cells by regulating mir-361-3p/socs3 axis. Int. J. Clin. Exp. Pathol..

[57] Wang G., Li Y., Peng Y., Tang J., Li H. (2018). Association of polymorphisms in malat1 with risk of coronary atherosclerotic heart disease in a chinese population. Lipids Health Dis..

[58] Lv F., Liu L., Feng Q., Yang X. (2021). Long non-coding rna malat1 and its target microrna-125b associate with disease risk, severity, and major adverse cardiovascular event of coronary heart disease. J. Clin. Lab. Anal..

[59] Wang H., Lin S., Yang Y., Zhao M., Li X., Zhang L. (2022). Significant role of long non-coding rna malat1 in deep vein thrombosis via the regulation of vascular endothelial cell physiology through the microrna-383-5p/bcl2l11 axis. Bioengineered.

[60] Hu W., Xin Y., Hu J., Sun Y., Zhao Y. (2019). Inhibitor of DNA binding in heart development and cardiovascular diseases. Cell. Commun. Signal..

[61] Cunningham T.J., Yu M.S., McKeithan W.L., Spiering S., Carrette F., Huang C.T., Bushway P.J., Tierney M., Albini S., Giacca M. (2017). Id genes are essential for early heart formation. Genes Dev..

[62] Kong D., He M., Yang L., Zhou R., Yan Y.-Q., Liang Y., Teng C.-B. (2019). Mir-17 and mir-19 cooperatively promote skeletal muscle cell differentiation. Cell. Mol. Life Sci..

[63] Qiu J., Li Y., Wang B., Sun X., Qian D., Ying Y., Zhou J. (2022). The role and research progress of inhibitor of differentiation 1 in atherosclerosis. DNA Cell Biol..

[64] Wang D., Wang D., Huang M., Zheng X., Shen Y., Fu B., Zhao H., Chen X., Peng P., Zhu Q. (2021). Transcriptomic characteristics and impaired immune function of patients who retest positive for SARS-CoV-2 rna. J. Mol. Cell Biol..

[65] Wei J., Shi Y., Zou C., Zhang H., Peng H., Wang S., Xia L., Yang Y., Zhang X., Liu J. (2022). Cellular id1 inhibits hepatitis b virus transcription by interacting with the novel covalently closed circular DNA-binding protein e2f4. Int. J. Biol. Sci..

[66] Luo Y., Wang G., Ren T., Zhang T., Chen H., Li Y., Yin X., Zhang Z., Sun Y. (2021). Screening of host genes regulated by id1 and id3 proteins during foot-and-mouth disease virus infection. Virus Res..

[67] Pattarabanjird T., Cress C., Nguyen A., Taylor A., Bekiranov S., McNamara C. (2020). A machine learning model utilizing a novel snp shows enhanced prediction of coronary artery disease severity. Genes.

[68] Valanti E.-K., Dalakoura-Karagkouni K., Fotakis P., Vafiadaki E., Mantzoros C.S., Chroni A., Zannis V., Kardassis D., Sanoudou D. (2022). Reconstituted hdl-apoe3 promotes endothelial cell migration through id1 and its downstream kinases erk1/2, akt and p38 mapk. Metab. Clin. Exp..

[69] Baccarelli A.A., Byun H.M. (2015). Platelet mitochondrial DNA methylation: A potential new marker of cardiovascular disease. Clin. Epigenet..

[70] Guarnieri J.W., Dybas J.M., Fazelinia H., Kim M.S., Frere J., Zhang Y., Albrecht Y.S., Murdock D.G., Angelin A., Singh L.N. (2022). Targeted down regulation of core mitochondrial genes during SARS-CoV-2 infection. bioRxiv.

[71] Hoque M.N., Khan M.A., Hossain M.A., Hasan M.I., Rahman M.H., Soliman M.E., Araf Y., Zheng C., Islam T. (2022). Differential gene expression profiling reveals potential biomarkers and pharmacological compounds against SARS-CoV-2: Insights from machine learning and bioinformatics approaches. bioRxiv.

[72] Guo C., Wang J., Jing L., Ma R., Liu X., Gao L., Cao L., Duan J., Zhou X., Li Y. (2018). Mitochondrial dysfunction, perturbations of mitochondrial dynamics and biogenesis involved in endothelial injury induced by silica nanoparticles. Environ. Pollut..

[73] Vermorken A.J.M., Zhu J., Holvoet P., Cui Y. (2021). The marker of cobalamin deficiency, plasma methylmalonic acid, may help identifying lysosomal iron trapping in patients. Its possible utility for heart failure. Redox Biol..

[74] Topol E.J. (2020). COVID-19 can affect the heart. Science.

[75] Zhong D., Jia-wei Z., Yan-an W. (2019). Current research progress of egfl7 in angiogenesis regulation. China J. Oral. Maxillofac. Surg..

[76] Heissig B., Salama Y., Takahashi S., Okumura K., Hattori K. (2021). The multifaceted roles of egfl7 in cancer and drug resistance. Cancers.

[77] Masoud A.G., Lin J., Azad A.K., Farhan M.A., Fischer C., Zhu L.F., Zhang H., Sis B., Kassiri Z., Moore R.B. (2020). Apelin directs endothelial cell differentiation and vascular repair following immune-mediated injury. J. Clin. Investig..

[78] Leng L., Cao R., Ma J., Mou D., Zhu Y., Li W., Lv L., Gao D., Zhang S., Gong F. (2020). Pathological features of COVID-19-associated lung injury: A preliminary proteomics report based on clinical samples. Signal. Transduct. Target. Ther..

[79] Sezer Zhmurov C., Timirci-Kahraman O., Amadou F.Z., Fazliogullari O., Basaran C., Catal T., Zeybek U., Bermek H. (2016). Expression of egfl7 and mirna-126-5p in symptomatic carotid artery disease. Genet. Test. Mol. Biomark..

[80] Li L., Zhao Y., Hu Y., Wang X., Jin Q., Zhao Y. (2022). Recombinant egfl7 mitigated pressure overload-induced cardiac remodeling by blocking pi3k γ /akt/ nfκb signaling in macrophages. Front. Pharmacol..

[81] Small E.M., Frost R.J., Olson E.N. (2010). Micrornas add a new dimension to cardiovascular disease. Circulation.

[82] Zhang M., Gu H., Xu W., Zhou X. (2016). Down-regulation of lncrna malat1 reduces cardiomyocyte apoptosis and improves left ventricular function in diabetic rats. Int. J. Cardiol..

[83] Hu H., Wu J., Yu X., Zhou J., Yu H., Ma L. (2019). Long non-coding rna malat1 enhances the apoptosis of cardiomyocytes through autophagy inhibition by regulating tsc2-mtor signaling. Biol. Res..

[84] Vausort M., Wagner D.R., Devaux Y. (2014). Long noncoding rnas in patients with acute myocardial infarction. Circ. Res..

[85] Puthanveetil P., Chen S., Feng B., Gautam A., Chakrabarti S. (2015). Long non-coding rna malat1 regulates hyperglycaemia induced inflammatory process in the endothelial cells. J. Cell. Mol. Med..

[86] Chen F., Li W., Zhang D., Fu Y., Yuan W., Luo G., Liu F., Luo J. (2022). Malat1 regulates hypertrophy of cardiomyocytes by modulating the mir-181a/hmgb2 pathway. Eur. J. Histochem..

[87] Stanley W.C., Recchia F.A., Lopaschuk G.D. (2005). Myocardial substrate metabolism in the normal and failing heart. Physiol. Rev..

[88] McCafferty C., Van Den Helm S., Letunica N., Attard C., Karlaftis V., Cai T., Praporski S., Swaney E., Burgner D., Neeland M. (2021). Increased platelet activation in SARS-CoV-2 infected non-hospitalised children and adults, and their household contacts. Br. J. Haematol..

[89] Dias S.S.G., Soares V.C., Ferreira A.C., Sacramento C.Q., Fintelman-Rodrigues N., Temerozo J.R., Teixeira L., Nunes da Silva M.A., Barreto E., Mattos M. (2020). Lipid droplets fuel SARS-CoV-2 replication and production of inflammatory mediators. PLoS Pathog..

[90] Glatz J.F.C., Wang F., Nabben M., Luiken J.J.F.P. (2021). Cd36 as a target for metabolic modulation therapy in cardiac disease. Expert Opin. Ther. Targets.

[91] Zhang X., Fan J., Li H., Chen C., Wang Y. (2021). Cd36 signaling in diabetic cardiomyopathy. Aging Dis..

[92] Shu H., Peng Y., Hang W., Zhou N., Wang D.W. (2021). Trimetazidine in heart failure. Front. Pharmacol..

[93] Bigotti M.G., Brancaccio A. (2021). High degree of conservation of the enzymes synthesizing the laminin-binding glycoepitope of α-dystroglycan. Open Biol..

[94] Ribeiro A.F., Souza L.S., Almeida C.F., Ishiba R., Fernandes S.A., Guerrieri D.A., Santos A.L., Onofre-Oliveira P.C., Vainzof M. (2019). Muscle satellite cells and impaired late stage regeneration in different murine models for muscular dystrophies. Sci. Rep..

[95] Joseph S., Schnicker N.J., Xu Z., Yang T., Hopkins J., Watkins M., Chakravarthy S., Davulcu O., Anderson M.E., Venzke D. (2022). Structure and mechanism of large1 matriglycan polymerase. bioRxiv.

[96] Katz M., Weinstein J., Eilon-Ashkenazy M., Gehring K., Cohen-Dvashi H., Elad N., Fleishman S.J., Diskin R. (2022). Structure and receptor recognition by the lassa virus spike complex. Nature.

[97] Saito F., Kanagawa M., Ikeda M., Hagiwara H., Masaki T., Ohkuma H., Katanosaka Y., Shimizu T., Sonoo M., Toda T. (2014). Overexpression of large suppresses muscle regeneration via down-regulation of insulin-like growth factor 1 and aggravates muscular dystrophy in mice. Hum. Mol. Genet..

[98] Kanagawa M. (2021). Dystroglycanopathy: From elucidation of molecular and pathological mechanisms to development of treatment methods. Int. J. Mol. Sci..

[99] D’Amario D., Amodeo A., Adorisio R., Tiziano F.D., Leone A.M., Perri G., Bruno P., Massetti M., Ferlini A., Pane M. (2017). A current approach to heart failure in duchenne muscular dystrophy. Heart.

[100] Hiess F., Detampel P., Nolla-Colomer C., Vallmitjana A., Ganguly A., Amrein M., Ter Keurs H.E., Benítez R., Hove-Madsen L., Chen S.W. (2018). Dynamic and irregular distribution of ryr2 clusters in the periphery of live ventricular myocytes. Biophys. J..

[101] Ferrantini C., Coppini R., Scellini B., Ferrara C., Pioner J.M., Mazzoni L., Priori S., Cerbai E., Tesi C., Poggesi C. (2016). R4496c ryr2 mutation impairs atrial and ventricular contractility. J. General. Physiol..

[102] Gallo Marin B., Aghagoli G., Lavine K., Yang L., Siff E.J., Chiang S.S., Salazar-Mather T.P., Dumenco L., Savaria M.C., Aung S.N. (2021). Predictors of COVID-19 severity: A literature review. Rev. Med. Virol..

[103] Wei J., Xu H., Shi L., Tong J., Zhang J. (2015). Trimetazidine protects cardiomyocytes against hypoxia-induced injury through ameliorates calcium homeostasis. Chem. -Biol. Interact..

[104] Reiken S., Sittenfeld L., Dridi H., Liu Y., Liu X., Marks A.R. (2022). Alzheimer’s-like signaling in brains of COVID-19 patients. Alzheimers Dement..

[105] Zeng Z., Huang N., Zhang Y., Wang Y., Su Y., Zhang H., An Y. (2020). Ctcf inhibits endoplasmic reticulum stress and apoptosis in cardiomyocytes by upregulating ryr2 via inhibiting s100a1. Life Sci..

[106] Kato T., Yamamoto T., Nakamura Y., Nanno T., Fukui G., Sufu Y., Hamada Y., Maeda T., Nishimura S., Ishiguchi H. (2017). Correction of impaired calmodulin binding to ryr2 as a novel therapy for lethal arrhythmia in the pressure-overloaded heart failure. Heart Rhythm..

[107] Acimovic I., Refaat M.M., Moreau A., Salykin A., Reiken S., Sleiman Y., Souidi M., Pribyl J., Kajava A.V., Richard S. (2018). Post-translational modifications and diastolic calcium leak associated to the novel ryr2-d3638a mutation lead to cpvt in patient-specific hipsc-derived cardiomyocytes. J. Clin. Med..

[108] Jansen J., Reimer K.C., Nagai J.S., Varghese F.S., Overheul G.J., de Beer M., Roverts R., Daviran D., Fermin L.A.S., Willemsen B. (2022). SARS-CoV-2 infects the human kidney and drives fibrosis in kidney organoids. Cell Stem Cell.

[109] Li S., Zhao F., Ye J., Li K., Wang Q., Du Z., Yue Q., Wang S., Wu Q., Chen H. (2022). Cellular metabolic basis of altered immunity in the lungs of patients with COVID-19. Med. Microbiol. Immunol..

[110] Iliev I.D., Cadwell K. (2021). Effects of intestinal fungi and viruses on immune responses and inflammatory bowel diseases. Gastroenterology.

[111] Liang X. (2022). Investigating the protective contribution of plcg2 p522r variant in microglia-mediated immune pathways in alzheimer’s disease. Alzheimer’s Dement..

[112] Shaath H., Vishnubalaji R., Elkord E., Alajez N.M. (2020). Single-cell transcriptome analysis highlights a role for neutrophils and inflammatory macrophages in the pathogenesis of severe COVID-19. Cells.

[113] Yang Q., Lin F., Wang Y., Zeng M., Luo M. (2021). Long noncoding rnas as emerging regulators of COVID-19. Front. Immunol..

[114] Huang K., Wang C., Vagts C., Raguveer V., Finn P.W., Perkins D.L. (2021). Long non-coding rnas (lncrnas) neat1 and malat1 are differentially expressed in severe COVID-19 patients: An integrated single cell analysis. medRxiv.

[115] Cremer S., Michalik K.M., Fischer A., Pfisterer L., Jae N., Winter C., Boon R.A., Muhly-Reinholz M., John D., Uchida S. (2019). Hematopoietic deficiency of the long noncoding rna malat1 promotes atherosclerosis and plaque inflammation. Circulation.

[116] McDonald J.T., Enguita F.J., Taylor D., Griffin R.J., Priebe W., Emmett M.R., Sajadi M.M., Harris A.D., Clement J., Dybas J.M. (2021). Role of mir-2392 in driving SARS-CoV-2 infection. Cell. Rep..

[117] Ait-Aissa K., Blaszak S.C., Beutner G., Tsaih S.W., Morgan G., Santos J.H., Flister M.J., Joyce D.L., Camara A.K.S., Gutterman D.D. (2019). Mitochondrial oxidative phosphorylation defect in the heart of subjects with coronary artery disease. Sci. Rep..

[118] Holvoet P., Vanhaverbeke M., Bloch K., Baatsen P., Sinnaeve P., Janssens S. (2016). Low mt-co1 in monocytes and microvesicles is associated with outcome in patients with coronary artery disease. J. Am. Heart Assoc..

[119] Gagliardi S., Poloni E.T., Pandini C., Garofalo M., Dragoni F., Medici V., Davin A., Visona S.D., Moretti M., Sproviero D. (2021). Detection of SARS-CoV-2 genome and whole transcriptome sequencing in frontal cortex of COVID-19 patients. Brain Behav. Immun..

[120] Wilkins S.E., Abboud M.I., Hancock R.L., Schofield C.J. (2016). Targeting protein-protein interactions in the hif system. ChemMedChem.

[121] Ma S., Sun S., Li J., Fan Y., Qu J., Sun L., Wang S., Zhang Y., Yang S., Liu Z. (2021). Single-cell transcriptomic atlas of primate cardiopulmonary aging. Cell Res..

[122] Aryankalayil M.J., Martello S., Bylicky M.A., Chopra S., May J.M., Shankardass A., MacMillan L., Sun L., Sanjak J., Vanpouille-Box C. (2021). Analysis of lncrna-mirna-mrna expression pattern in heart tissue after total body radiation in a mouse model. J. Transl. Med..

[123] Saha C., Laha S., Chatterjee R., Bhattacharyya N.P. (2021). Co-regulation of protein coding genes by transcription factor and long non-coding rna in SARS-CoV-2 infected cells: An in silico analysis. Non-Coding RNA.

[124] Zheng J., Tan Q., Chen H., Chen K., Wang H., Chen Z., Xi Y., Yin H., Lai K., Liu Y. (2021). Lncrnasnhg7003 inhibits the proliferation, migration and invasion of vascular smooth muscle cells by targeting the mir13065p/sirt7 signaling pathway. Int. J. Mol. Med..

[125] Pan Z., Fan Z., Ma J., Liu H., Shen L., He B., Zhang M. (2019). Profiling and functional characterization of circulation lncrnas that are associated with coronary atherosclerotic plaque stability. Am. J. Transl. Res..

[126] Kyei F., Asante D.-B., Edekor J.A.M., Sarpong E., Gavor E., Konja D. (2016). Down-regulation of id1 and id3 genes affects growth and survival of human umbilical vein endothelial cells (huvecs). J. Appl. Biol. Biotechnol..

[127] Zhang N., Subbaramaiah K., Yantiss R.K., Zhou X.K., Chin Y., Scherl E.J., Bosworth B.P., Benezra R., Dannenberg A.J. (2015). Id1 expression in endothelial cells of the colon is required for normal response to injury. Am. J. Pathol..

[128] Nasser M.I., Masood M., Adlat S., Gang D., Zhu S., Li G., Li N., Chen J., Zhu P. (2021). Mesenchymal stem cell-derived exosome microrna as therapy for cardiac ischemic injury. Biomed. Pharmacother..

[129] Huang X., Qu R., Ouyang J., Zhong S., Dai J. (2020). An overview of the cytoskeleton-associated role of pdlim5. Front. Physiol..

[130] Huang J., Cai C., Zheng T., Wu X., Wang D., Zhang K., Xu B., Yan R., Gong H., Zhang J. (2020). Endothelial scaffolding protein enh (enigma homolog protein) promotes phlpp2 (pleckstrin homology domain and leucine-rich repeat protein phosphatase 2)-mediated dephosphorylation of akt1 and enos (endothelial no synthase) promoting vascular remodeling. Arterioscler. Thromb. Vasc. Biol..

[131] Green I.E., Williams S.R., Sale M.M., Keene K.L., Worrall B.B., Southerland A.M. (2020). Differential expression of phactr1 in atheromatous versus normal carotid artery tissue. J. Clin. Neurosci..

[132] Zhang Z., Jiang F., Zeng L., Wang X., Tu S. (2018). Phactr1 regulates oxidative stress and inflammation to coronary artery endothelial cells via interaction with nf-kappab/p65. Atherosclerosis.

[133] Adlam D., Olson T.M., Combaret N., Kovacic J.C., Iismaa S.E., Al-Hussaini A., O’Byrne M.M., Bouajila S., Georges A., Mishra K. (2019). Association of the phactr1/edn1 genetic locus with spontaneous coronary artery dissection. J. Am. Coll. Cardiol..

[134] Kanduc D. (2021). Anti-SARS-CoV-2 immune response and sudden death: Titin as a link. Adv. Stud. Biol..

[135] Itoh-Satoh M., Hayashi T., Nishi H., Koga Y., Arimura T., Koyanagi T., Takahashi M., Hohda S., Ueda K., Nouchi T. (2002). Titin mutations as the molecular basis for dilated cardiomyopathy. Biochem. Biophys. Res. Commun..

[136] Zaunbrecher R.J., Abel A.N., Beussman K., Leonard A., von Frieling-Salewsky M., Fields P.A., Pabon L., Reinecke H., Yang X., Macadangdang J. (2019). Cronos titin is expressed in human cardiomyocytes and necessary for normal sarcomere function. Circulation.

[137] Zolfaghari Emameh R., Nosrati H., Eftekhari M., Falak R., Khoshmirsafa M. (2020). Expansion of single cell transcriptomics data of sars-cov infection in human bronchial epithelial cells to COVID-19. Biol. Proced. Online.

[138] Wang Z., Grange M., Pospich S., Wagner T., Kho A.L., Gautel M., Raunser S. (2022). Structures from intact myofibrils reveal mechanism of thin filament regulation through nebulin. Science.

[139] Haslbauer J.D., Zinner C., Stalder A.K., Schneeberger J., Menter T., Bassetti S., Mertz K.D., Went P., Matter M.S., Tzankov A. (2021). Vascular damage, thromboinflammation, plasmablast activation, t-cell dysregulation and pathological histiocytic response in pulmonary draining lymph nodes of COVID-19. Front. Immunol..

[140] Oakley R.H., Cidlowski J.A. (2015). Glucocorticoid signaling in the heart: A cardiomyocyte perspective. J. Steroid Biochem. Mol. Biol..

[141] Zhao Q., Wu K., Li N., Li Z., Jin F. (2018). Identification of potentially relevant genes for myocardial infarction using rna sequencing data analysis. Exp. Ther. Med..

